# Infection prevention and control in Swedish nursing homes for older adults before and after the COVID-19 pandemic: A descriptive study

**DOI:** 10.1177/17571774251394896

**Published:** 2025-10-31

**Authors:** Susanne Wiklund, Christina Stamm, Ann Tammelin

**Affiliations:** 1 Department of Medicine, Solna (MedS), Division of Infectious Diseases, 27106 Karolinska Institutet, Stockholm, Sweden; 2 Department of Infection Control and Hospital Hygiene, Stockholm County Council, Stockholm, Sweden

**Keywords:** nursing homes, COVID-19 pandemic, infection control and prevention

## Abstract

**Background:**

In early spring 2020, the spread of SARS-CoV-2, began which developed into a global pandemic of the disease COVID-19. Nursing homes (NHs) for older adults in Sweden experienced a significant spread of the virus resulting in many deaths among the residents and cases of illness among the employees.

**Aim:**

To explore how supply and use of products for protection of residents and staff against infection as well as IPC (infection prevention and control) training changed in Swedish NHs for older adults between the years 2019 and 2023.

**Methods:**

A web-based survey form with questions concerning supply and use of products as well as training of employees in IPC and use of governing and supporting documents concerning IPC in 2019 and 2023 was sent out to 300 NHs in Stockholm County in January 2024. This was the total number of NHs which were in operation both in 2019 and 2023.

**Results:**

Answers were obtained from 130 NHs with a total of 7377 residents. The response rate was 43.3%. Supply of all products was improved in 2023. There was a significant association between improved supply and improved use for all products (p < .01) except for single use gloves. The number of employees who received training on IPC had risen significantly from 2019 to 2023 (p < .01). In 2023, all respondents used guiding documents on IPC.

**Discussion:**

The COVID-19-pandemic resulted in several improvements concerning IPC in NHs for older adults in Sweden. After the pandemic staff got better training, managers used governing and supporting documents to a higher degree and the supply and use of protective products was improved, except for the use of single use gloves. The difficulties in correct use of gloves were found in 2019 and remained in 2023.

## Background

In Sweden, the 290 municipalities are legally obligated to meet the need for social service and housing of older adults above the age of 65. A place in a nursing home (NH) is provided by the municipality if the needs for assistance in daily life are assessed as too high for home help service. In 2023, 16.6 % of Swedish persons above 85 years of age lived in NHs (National Board of Health and Welfare, 2024). Residence in a NH includes daily care as help with personal hygiene, dressing and eating which is provided by assistant nurses. The residents have single rooms with separate bathrooms and there are also shared areas for socializing. The person can live in a NH for the rest of her/his life, even if needs for care changes (World Health Organization, 2022). When assisting with personal hygiene, microorganisms can be transferred between staff and care recipients which increases the risk for infection. Transfer can also occur indirectly through contaminated surfaces and objects in shared facilities such as dining rooms and living rooms (Kanamori et al., 2023; Martinez-Payá et al., 2022; Wang et al., 2020).

Sweden has had a Regulation on Basic Hygiene in health care and care for elderly since 2007 (National Board of Health and Welfare, 2015). How and when single use gloves, single use aprons and alcohol-based hand disinfectant should be used is described in this regulation. The aim is to protect patients in healthcare and residents in NHs from microorganisms transferred by contact.

The use of medical face masks, face shields and respiratory protective devices is regulated by Regulation on the Use of Personal Protective Equipment launched by Swedish Work Environment Authority in 2001 (Swedish Work Environment Authority, 2001). The aim of the regulation is to protect the employee from infection caused by splash to the face or by inhalation.

In early spring 2020 the spread of SARS-CoV-2, began which developed into a global pandemic of the disease COVID-19. NHs for older adults in Stockholm County experienced a significant spread of the virus resulting in many deaths among the residents and cases of illness among the employees. Scientific articles have been written about the experiences of staff during the pandemic, as well as the vulnerable situation of managers of NHs (Behrens and Naylor, 2020; Bergqvist et al., 2023; Liljas et al., 2024; Lövenmark and Marmstål Hammar, 2024). The Swedish Health and Social Care Inspectorate (IVO) and The National Board of Health and Welfare have reported on deficiencies in care in the NHs during the pandemic (The Health and Social Care Inspectorate, 2023). However, none of these articles and reports address the prerequisites for preventing transmission and/or infection. Reports address the prerequisites for preventing transmission and/or infection. This study was conducted to investigate if the capability regarding such prevention was changed by the pandemic.

## Aim

The aim of this study was to explore how supply and use of products for protection of residents and staff as well as training concerning IPC was changed in Swedish NHs for older adults between the years 2019 and 2023.

## Methods

### Setting and participants

There were 305 NHs for older adults, totaling 15,700 residents across Stockholm County. (National Board of Health and Welfare, 2024). Five of these opened after 2019, so they were excluded from this study.

### Data collection

A letter with information about the study was sent from the responsible researcher to the manager of each of the included 300 NHs in January 2024. Of the included NHs, 166 (55.3%) were run by private companies and 134 (44.7%) by the municipalities. The researchers constructed the questionnaire constructed the questionnaire consisting of 66 questions. The questions were close-ended with fixed alternatives for answers but also a possibility of giving comments on each question. These questions were transformed to a web-based survey form by a commercial company (Enkätfabriken, Goteborg, Sweden) which was responsible for sending it out to the persons who had received the information letter and for collecting the answers. This secured that the identities of the respondents were unknown to the researchers. The compiled results were then sent to the researchers as two Excel spreadsheets. The study was performed between 23th of January and the 1st of March 2024. The survey form was sent out by email with a total of five reminders. The number of answers rose from 17 after the first dispatch of the survey to 130 after the last reminder.

The questions sent to the managers concerned number of employees caring for the older adults (including full and part time employees), number of residents, supply and use of single use gloves and aprons, alcohol-based hand disinfectant, medical face masks, face shields and respiratory protective devices in 2019 and whether the supply and use was improved, unchanged or deteriorated in 2023. Questions were also asked about training of the staff in IPC and about the managers access to governing and supporting documents. The instructions to the managers were that all staff included in the care of elderly should be included in the count of employees. The terms used in the questionnaire were well-known to everybody working in nursing homes for older adults.

### Data analysis

Data analysis was performed with Epi Info^TM^ (CDC) version 7.2.2.6. Fisher Exact Test 2-tailed was used for analysis. A *p*-value of <.05 was regarded as significant.

## Results

### Study population

Answers were obtained from 130 NHs with a total of 7377 residents (3769 in private NH and 3608 in NHs run by the municipalities) and 9412 employees (4817 in private NHs and 4595 in NHs run by the municipalities). The total response rate was 43.3% (130/300), 44.0% (73/166) among the private NHs and 42.5% (57/134) among those run by the municipalities. All surveys returned were accepted for inclusion. Complete answers were obtained from 101/130 (77.7%) NHs. The proportion of complete answers was 76.7% (56/73) from private NHs and 78.9% (45/57) from NHs run by municipalities. From the respondents with uncomplete answers single questions were answered to a variable extent.

### Number of residents

The number of residents varied from 9 to 108 in the private NHs (median 54, average 52) and from 18 to 174 (median 57.5, average 64) in those run by the municipalities.

### Staffing

The number of employees taking part in the care of the older adults was on average 1.3 per resident both in the private NHs (range 0.7–2.8, median 1.3) and those run by the municipalities (range 0.2–2.8, median 1.3).

### Supply and use of products for protection of residents and staff concerning IPC

In 2019 the supply of single use gloves, single use aprons and alcohol-based hand disinfectant was regarded as sufficient by 88%–92% of the respondents. (Table 1).

**Table 1.** Situation in 2019. Supply and Correct Use of Products. Proportion (%) and Number Among Answering Nursing Homes (*n*/*n*).

	Sufficient supply	Correct use among all	Correct use among those with sufficient supply	Correct use among those with insufficient supply	Significance of association between sufficient supply and correct use, *p*
Single use gloves^a^	90.8 (108/119)	84.9 (101/119)	86.1 (93/108)	72.7 (8/11)	0.36
p^b^	90.9 (60/66)	80.3 (53/66)	81.7 (49/60)	66.7 (4/6)	0.34
m^c^	90.6 (48/53) ns	90.6 (48/53) ns	91.7 (44/48) ns	80.0 (4/5) ns	0.40
Single use aprons^a^	88.5 (108/122)	94.3 (115/122)	97.2 (105/108)	71.4 (10/14)	<0.01
p^b^	88.4 (61/69)	89.9 (62/69)	95.1 (58/61)	50.0 (4/8)	<0.01 ns
m^c^	88.7 (47/53) ns	100.0 (53/53) *p =* .*02*	100.0 (47/47) ns	100.0 (6/6) ns	
Alcohol-based hand disinfectant^a^	92.0 (104/113)	89.4 (101/113)	92.3 (96/104)	55.6 (5/9)	<0.01 ns
p^b^	93.5 (58/62)	85.5 (53/62)	87.9 (51/58)	50.0 (2/4)	0.02
m^c^	90.2 (46/51) ns	94.1 (48/51) ns	97.8 (45/46) ns	60.0 (3/5) ns	
Medical face masks^d^	63.6 (75/118)	79.7 (94/118)	93.3 (70/75)	55.8 (24/43)	<0.01
p^b^	72.3 (47/65)	76.9 (50/65)	91.5 (43/47)	38.9 (7/18)	<0.01
m^c^	52.8 (28/53) *p =* .*04*	83.0 (44/53) ns	96.4 (27/28) ns	68.0 (17/25) ns	<0.01
Face shields^d^	62.1 (72/116)	80.2 (93/116)	95.8 (69/72)	54.5 (24/44)	<0.01
p^b^	60.9 (40/64)	76.6 (49/64)	95.0 (38/40)	45.8 (11/24)	<0.01
m^c^	61.5 (32/52) ns	84.6 (44/52) ns	96.9 (31/32) ns	65.0 (13/20) ns	<0.01
Respiratory protective devices^d^	54.4 (62/114)	71.1 (81/114)	98.4 (61/62)	38.5 (20/52)	<0.01
p^b^	56.5 (35/62)	67.7 (42/62)	97.1 (34/35)	29.6 (8/27)	<0.01
m^c^	51.9 (27/52) ns	75.0 (39/52) ns	100.0 (27/27) ns	48.0 (12/25) ns	<0.01

^a^Supply was regarded as sufficient to be able to follow the Regulation on basic hygiene (SOSFS 2015:10).

^b^Private nursing home.

^c^Municipality nursing home.

^d^Supply was regarded as sufficient to be able to follow regional guidelines.

ns = not significant difference.

The supply of medical face masks, face shields and respiratory protective device was regarded as sufficient in 2019 by 54%–63% of the respondents. For medical face masks there was a significant difference (*p* = .04) between the private NHs and the municipality NHs (Table 1).

In 2019 there was a significant association between sufficient supply and correct use of all protective products except for single use gloves. The lack of association for single use gloves was found both in NHs run by the municipalities and in private NHs (Table 1).

The supply of all kinds of products was improved in 2023 compared to 2019 both in NHs run by the municipalities and in private NHs.

In 2023 correct use of single use gloves, single use aprons and alcohol-based hand disinfectant was improved in 42%–52% of the NHs. There was a significant association between improved supply and improved use for all products except for single use gloves (Table 2).

**Table 2.** Situation in 2023. Supply and Correct Use of Products. Proportion (%) and Number Among Answering Nursing Homes (*n*/*n*).

	Supply of the product was improved, % (*n*)	Correct use of the product was improved, % (*n*)	Improved use among those where the supply was improved, % (*n*)	Improved use among those where the supply was not improved, % (*n*)	Significance of association between improved supply and improved use, *p*
Single use gloves^a^	48.7 (58/119)	42.0 (50/119)	53.4 (31/58)	31.1 (19/61)	0.02
p^b^	56.1 (37/66)	40.9 (27/66)	54.1 (20/37)	24.1 (7/29)	0.02 ns
m^c^	39.6 (21/53) ns	43.4 (23/53) ns	52.4 (11/21) ns	37.5 (12/32) ns	
Single use aprons^a^	50.8 (62/122)	47.5 (58/122)	66.1 (41/62)	28.3 (17/60)	<0.01
p^b^	53.6 (37/69)	47.8 (33/69)	64.9 (24/37)	28.1 (9/32)	<0.01
m^c^	47.2 (25/53) ns	47.2 (25/53) ns	68.0 (17/25) ns	28.6 (8/28) ns	<0.01
Alcohol-based hand disinfectant^a^	55.8 (63/113)	52.2 (59/113)	63.5 (40/63)	38.0 (19/50)	<0.01 ns
p^b^	56.5 (35/62)	43.5 (27/62)	54.3 (19/35)	29.6 (8/27)	ns
m^c^	54.9 (28/51) ns	62.7 (32/51) ns	77.8 (21/28) ns	47.8 (11/23) ns	
Medical face masks^d^	75.4 (89/118)	61.9 (73/118)	71.9 (64/89)	31.0 (9/29)	<0.01
p^b^	75.4 (49/65)	56.9 (37/65)	65.3 (32/49)	31.3 (5/16)	0.02
m^c^	75.5 (40/53) ns	67.9 (36/53) ns	80.0 (32/40) ns	30.8 (4/13) ns	<0.01
Face shields^d^	69.8 (81/116)	53.4 (62/116)	67.9 (55/81)	20.0 (7/35)	<0.01
p^b^	62.5 (40/64)	46.9 (30/64)	62.5 (25/40)	20.8 (5/24)	<0.01
m^c^	78.8 (41/52) ns	61.5 (32/52) ns	73.2 (30/41) ns	18.2 (2/11) ns	<0.01
Respiratory protective devices^d^	66.7 (76/114)	57.9 (66/114)	78.9 (60/76)	15.8 (6/38)	<0.01
p^b^	58.1 (36/62)	46.8 (29/62)	72.2 (26/36)	11.5 (3/26)	<0.01
m^c^	76.9 (40/52) *p =* .*05*	71.2 (37/52) *p =* .*01*	85.0 (34/40) ns	25.0 (3/12) ns	<0.01

^a^Supply was regarded as sufficient to be able to follow the Regulation on basic hygiene (SOSFS 2015:10).

^b^Private nursing home.

^c^Municipality nursing home.

^d^Supply was regarded as sufficient to be able to follow regional guidelines.

ns = not significant difference.

In 2023 correct use of medical face masks, face shields and respiratory protective device was improved in 53%–62% of all the NHs. Improvement for use of respiratory protective device was significantly higher in the NHs run by the municipalities than in the private NHs (p = .05). Correct use was improved in 68%–79% of the NHs where supply was improved. The improvement was significantly higher among the NHs run by the municipalities (p = .01). The association between improved supply and improved use was significant for all products (Table 2).

### Comments from respondents concerning supply and use of products

Single use gloves.

(1) In 2019 single use gloves were used everywhere even when not needed.
(2) There has been continuous education to secure correct use of single use gloves.

Single use aprons.

(3) All staff was educated about how to use single use aprons before the pandemic.

Alcohol-based hand disinfectant.

(4) Many forget to disinfect their hands before performing care.

Medical face masks.

(5) Before the COVID-19 pandemic medical face masks were hardly used at all.

Face shields.

(6) We did not use face shields before the pandemic.

Respiratory protective devices.

(7) Respiratory protective devices were not used in NHs in 2019

### IPC training for the staff

Of the respondents 78.9% (86/109) answered that new employees participated in mandatory introductory training concerning IPC in 2019. The corresponding figure for 2023 was 93.6% (102/109). The improvement was statistically significant (*p* < .01) (Table 3).

**Table 3.** Introductory and Repetitive Training on IPC. Proportion (%) and Number Among Answering Nursing Homes (*n*/*n*).

	2019	2023	Significance of change, *p*
New employees took part in introductory training	78.9 (86/109)	93.6 (102/109)	0.002
p^a^	80.0 (48/60)	96.7 (58/60)	0.008
m^b^	77.6 (38/49) ns	89.8 (44/49) ns	ns
Employees took part in repetitive education	77.5 (79/102)	92.2 (94/102)	0.005
p^a^	75.0 (42/56)	92.9 (52/56)	0.02
m^b^	80.4 (37/46) ns	91.3 (42/46) ns	ns
Employees took part in both introductory training and repetitive education	64.2 (70/86)	81.7 (89/102)	ns
p^a^	79.2 (38/48)	87.9 (51/58)	ns
m^b^	84.2 (32/38) ns	86.4 (38/44) ns	ns

^a^Private nursing home

^b^Municipality nursing home

ns = not significant difference.

Face-to-face training was used by 75.5% (65/86) in 2019 and 71.5% (73/102) in 2023. Digital learning was used by 81.3% (70/86) in 2019 and 89.2% (91/102) in 2023.

Participation in introductory training was higher in NHs run by private companies than in NHs run by municipalities both in 2019 and 2023 (Table 3).

Of the respondents 77.5% (79/102) answered that employees received repetitive training concerning IPC in 2019 and 92.2% (94/102) in 2023. The change was significant (p < .01) (Table 3). Face-to-face training was used by 70.9% (56/79) in 2019 and 63.8% (60/94) in 2023. Digital learning was used by 88.6% (70/79) in 2019 and 95.7% (90/94) in 2023.

### Comments from respondents about introductory training

In 2019,

(1) We had briefings at workplace meetings and introductions. There was also a written routine that everyone signed and took part in upon new employment.

In 2023,

(2) The training we have for new employees has been developed after the pandemic.

### Comments from respondents about repetitive training

In 2019,

(1) Annual digital training through the Department of Infection Control and Hospital Hygiene in Stockholm County

In 2023

(2) Our Local Authority Senior Medical Adviser initiates self-assessment of Basic hygiene routines regularly.

## Access to governing and supporting documents

Of the respondents 92.1% (93/101) answered that they were aware of the governing and supporting documents concerning IPC in 2019. Of those, 87.1% (81/93) said that the documents were used in daily work. For 2023 all respondents claimed that they used such documents.

### Comments from respondents who used the documents in 2019

(1) Most of these documents can be found in our management system and in local procedures.

### Comments from respondents who did not use the documents in 2019

(2) It wasn’t relevant for us managers to read all this before the pandemic.

## Discussion

In a previous Swedish study by Bergqvist et al. the staff described inconsistent guidelines and the absence of leadership during the pandemic. The importance of good leadership during the pandemic is described (Bergqvist et al., 2023). To maintain good hygienic standard, governing and guiding documents are needed. The results in this study show that in 2019 92% of managers were aware of the availability of guiding and supporting documents but only 87% of those used them in their daily work. It is remarkable that so few of the managers were unaware of or did not use the documents available in 2019 as there is a legal demand for good hygienic standard. The figures for 2023 shows that the pandemic made managers aware of their responsibility to comply with them.

Previous studies have described how important it is for staff in nursing homes to receive continuous training about IPC (Bergqvist et al., 2023; Liljas et al., 2024; Lövenmark and Marmstål Hammar, 2024). A Norwegian study by Ree et al. described the importance of training in infection control (Ree et al., 2023). From the results, we see that when it comes to introductory training of newly employed staff on the topic of IPC, only in 79% of the NHs staff underwent such training in 2019. The number receiving introductory training had increased to almost 94% in 2023 which was statistically significant. One interpretation could be that during the pandemic, the importance of training in IPC was recognized., When it comes to repetitive training on IPC for existing employees, the 2019 results show that 77.5% received it, and that the figure increased significantly to 92.2% in 2023. The form for training changed from face-to-face to digital between 2019 and 2023. In 2023, around 90% of the NHs used digital training for introduction and almost 96% for repetitive training. One reason may be that the Department of Infection Control and Hospital Hygiene in Stockholm County today provides several digital trainings on the subject IPC.

Before the COVID-19 pandemic the supply of products aimed to protect residents in NHs from contact transmission of microorganisms was good as they were available in around 90% of the NHs with no significant difference between private NHs and those run by the municipalities. During the pandemic the necessity of protecting the elderly from infection was highlighted both through media and through inspection of the NHs by authorities. This contributed to efforts made to secure supply after the pandemic. This is shown as 50%–56% of all NHs say that supply of single use gloves, single use aprons and alcohol-based hand disinfectant was improved in 2023 and even more improved (93%–100%) in those with an insufficient supply before the pandemic.

Correct use of single use gloves was inferior to the correct use of single use aprons and hand disinfectant in 2019. Single use glove was the only product without significant association between sufficient supply and correct use. According to the regulation SOSFS 2015:10 “gloves must be used if there is a risk that hands will come into contact with body fluids during a health care or care task.” Gloves should not be used in other situations. Incorrect use of gloves has been shown in several studies performed both in NHs and in acute care (Jain et al., 2019; Loveday et al., 2014). Gloves are widely overused, for example, when making clean beds, when serving meals and when assisting a person with putting on clothes. Inappropriate use might lead to spread of microorganisms (Loveday et al., 2014; Patterson et al., 2017). The improvement of correct use of gloves from 2019 to 2023 was unfortunately modest and lower than for the other products both in the total cohort of NHs and in those where the supply was improved. The figures show that improved use is not only a matter of improved supply.

In 2019 the supply of products aimed at personal protection of staff was lower than the supply of products aimed at protection of the residents. This might be a result of several years of hard work to implement the regulation on Basic hygiene and thus not emphasize staff protection as much. It seems however that the staff had knowledge about correct use of personal protective equipment (PPE) when it was in place. The supply of PPE was improved between 2019 and 2023 as well as the correct use. Tsang et al. has identified lack of availability as the most common barrier to PPE use, i.e., if PPE is at hand the staff is willing to use it which is supported by our results (Tsang et al., 2023; Van Belle et al., 2024).

## Limitations

A limitation of this study is that the response rate only was around 43%. The answering NHs, however, covered 47% of the total number of residents in NHs in Stockholm County. There is no reason to believe that the situation was different among the other NHs. Another limitation is that the study was made in 2024 which could make the answers concerning the situation in 2019 less reliable. Direct observation of staff behaviors by an IPC-nurse might have added to the reliability of the managers answers but was not possible to perform in 130 NHs.

## Conclusions

The COVID-19-pandemic resulted in several improvements concerning IPC in NHs for older adults in Sweden. After the pandemic staff got better training, managers used governing and supporting documents to a higher degree and the supply and use of protective products was improved, except for the use of single use gloves. The difficulties in correct use of gloves were found in 2019 and remained in 2023.
